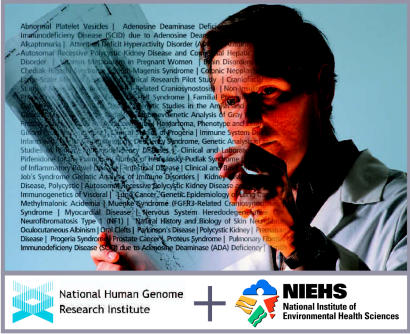# Human Genes and the Environment Research Training Program

**Published:** 2007-04

The National Institute of Environmental Health Sciences (NIEHS) and the National Human Genome Research Institute (NHGRI) announce the new Human Genes and the Environment Research Training Program, a component of the NIH Genes and the Environment Initiative, a multi-institute program aimed at identifying the genetic and environmental underpinnings of common illnesses.

The goal of this new training program is to produce a new generation of researchers who are equally at home in genomics and environmental health sciences and can interact seamlessly with scientists from both disciplines. This cadre of new researchers not only will be equipped to advance methodologies and technologies in environmental genomics/genetics, but also will use these tools and resources to disentangle and evaluate the enormous number of environmental factors that directly influence or interact with genotypes, resulting in phenotypic expression and clinical or physiologic endpoints.

Applications responsive to the Human Genes and the Environment Research Training Program announcement are to support trainees at the predoctoral and postdoctoral level and may include short-term training experiences. Inclusion of physicians and other clinically trained individuals at all levels of the program is encouraged. Training opportunities are to be structured so that each trainee is supervised by a mentoring team of at least two mentors, one of whom has expertise in the relevant environmental exposure or exposure biology aspect of the research and the other having relevant genetic/genomic expertise. The mentoring team should also be structured to provide expertise and guidance in the relevant clinical or disease end point of the research so that trainees can be fully aware of the impact of their research on the these ultimate end points.

For more information on this new training program see: **http://grants.nih.gov/grants/guide/rfa-files/RFA-ES-07-002.html**

Applications submitted in response to this program announcement will be assigned to either the NIEHS or the NHGRI for funding purposes, but will be considered a programmatic unit for purposes of scientific administration and coordination.

Programs to be assigned to the NIEHS will also need to conform to the Restructuring Notice NOT-ES-06-007 (http://grants1.nih.gov/grants/guide/notice-files/NOT-ES-06-007.html)

Contact

**Carol Shreffler, Ph.D.** | shreffl1@niehs.nih.gov

## Figures and Tables

**Figure:**